# Fever and Ague Annihilated

**Published:** 1859-03

**Authors:** 


### FEVER AND AGUE ANNIHILATED.

In a series of letters now in course of publication, "Trip to the Rappahannock," No. seven, in that inimitable paper, The Home Journal, the writer makes an "eminent" Virginia doctor say, that the avoidance of inhaling the out-door morning and evening air, is a certain means of exterminating fever and ague, called elsewhere chills and fevers. We advocated that doctrine a quarter of a century ago, and every year for five years past, in "Hall's Journal of Health." On pages 32, and 217 of vol. 5, 1858, the subject is treated, the declarations made, and the reasons given in the plainest manner possible. It seems further, that the eminent writer of the Rappahannock letters has been deluged with enquiries as to further particu lars, leading him to suppose that the information was a knowledge much wanted.

